# The physico-chemical properties and biostimulative activities of humic substances regenerated from lignite

**DOI:** 10.1186/2193-1801-3-156

**Published:** 2014-03-21

**Authors:** Jan David, Daniela Šmejkalová, Šárka Hudecová, Oldřich Zmeškal, Ray von Wandruszka, Tomáš Gregor, Jiří Kučerík

**Affiliations:** Faculty of Chemistry, Institute of Physical and Applied Chemistry and Materials Research Centre, Brno University of Technology, Purkyňova 464/118, CZ–612 00 Brno, Czech Republic; Contipro Pharma, Dolní Dobrouč 401, CZ–561 02 Dolní Dobrouč, Czech Republic; Faculty of Mathematics and Physics, Department of Probability and Mathematical Statistics, Charles University in Prague, Sokolovská 83, CZ–186 75 Prague 8, Czech Republic; College of Science, Department of Chemistry, University of Idaho, 804 South Rayburn Street, Renfrew Hall Rm 116, PO Box 442343, Moscow, 83844-2343 ID USA; Faculty of Agronomy, Department of Food Technology, Mendel University in Brno, Zemědělská 1, CZ–613 00 Brno, Czech Republic; Institute of Environmental Sciences, Soil and Environmental Chemistry, University of Koblenz-Landau, Fortstrasse 7, D–76829 Landau, Germany

**Keywords:** Lignite humic substances, Modification, Thermal analysis, Chromatography, Spectroscopy, Biological activity

## Abstract

The positive effect of humic acids on the growth of plant roots is well known, however, the mechanisms and role of their physical structure in these processes have not been fully explained yet. In this work, South-Moravian lignite was oxidized by means of nitric acid and hydrogen peroxide to produce a set of regenerated humic acids. The elemental composition, solid state stability and solution characteristics were determined and correlated *in vitro* with their biological activity. A modified hydroponic method was applied to determine the effects of their potassium salts on *Zea mays* seedlings roots with respect to the plant weight, root length, root division, and starch and protein content. The relations between the determined parameters were evaluated through Principal Component Analysis and Pearson’s correlation coefficients. The results indicated that the most important factor determining the biological activity of South-Moravian lignite potassium humates is related to the nature of self-assemblies, while the chemical composition had no direct connection with the root growth of *Zea mays* seedlings. It was demonstrated a controlled processing that provided humic substances with different chemical and physicochemical properties and variable biological activity.

## Introduction

Humic substances (humics, HS) are refractory, dark-colored heterogeneous organic compounds produced in the decay of the total biota in the environment (Stevenson 1994). From the chemical point of view, they are a heterogeneous mixture of fragments of lignins, cellulose, suberins and cutins. Their unique structure makes them a versatile material with applications in industry, medicine, environmental protection, and agriculture. It is becoming clearer, that the presence of humics in soil is necessary for sustainable agriculture, due to their ability to condition the soil, enhance its stability and increase its resistance to erosion (Laker et al. 1993; Spaccini et al. 2002), ensure enhanced biological activity (Canellas et al. 2002; Canellas et al. 2008; Nardi et al. 2000a, b; Zandonadi et al. 2007) and obtain higher crop yields (Antošová et al. 2008; Brownell et al. 1987; Eyheraguibel et al. 2008). In addition, humics have the ability to sequestrate soil pollutants (Evdokimov and von Wandruszka 1998; Sanjay et al. 1999; Senesi and Loffredo 2001; von Wandruszka 2000), and may be used in soil remediation (Fava et al. 2004; Stehlíčková et al. 2009; von Wandruszka 2000).

The biological activity of humics has been recognized in 1917 (Bottomley 1917), while their hormone–like nature was reported later (Canellas et al. 2008; Nardi et al. 2002). Aguirre et al. (2009) noted that the biological activity of humics is based on their ability to promote the expression of selected genes encoding enzymes like Fe^III^ chelate reductase, plasma membrane H^+^ATPase, and Fe^II^ high affinity transporter. Mora et al. (2010) questioned this “hormone-like” idea, since no cytokinins, gibberellins and indolacetic acid were found in humic acid samples. Instead, they hypothesized that humics enhance the activation of root plasma membrane H^+^ATPase, since this may cause significant changes in the root-to-shoot distribution of NO_3_^–^ and therefore of cytokinins and polyamines.

Piccolo et al. (1992) showed that the humic fraction with the highest acid functionality and the smallest molecular size had the greatest effect on plant nitrate uptake and hormone-like activity. In contrast, neither the aliphatic nor the aromatic content of the extracts appeared to play a role in the biological activity (Nardi et al. 2000a, b, and 2002) confirmed those observations and concluded that the smaller molecular size fractions can be partially taken up by the plasmolemma of higher plant cells, whereas the larger fractions (> 3.5 kDa) interacted only with the cell walls. In contrast, Canellas et al. (2010) showed that the size fractions of vermicompost humics obtained by preparative HPSEC (High performance size exclusion chromatography) had similar biological activities. In the work of Vlčková et al. (2009), the highest biological activity of humics toward *Zea mays* (tested by the method of Antošová et al. (2008)), was observed for 35–175 kDa molecular weight fraction.

Lignite, *i.e.* low rank coal, has been recognized as a valuable source of humics (Kučerík et al. 2003). The content of humics can be increased by regeneration processes with nitric acid, potassium manganate (VII), sulfuric acid, or hydrogen peroxide (Berkowitz 1985, Rausa et al. 1994;Kučerík et al. 2003;Vlčková et al. 2009). However, understanding the influence of lignite regeneration on the physicochemical and biological behavior is still incomplete. The same holds true for optimization of the regeneration processes (e.g. regeneration agent, type and concentration, and regeneration time). Since regeneration is most effective in suspension with nitric acid or hydrogen peroxide (Kučerík et al. 2008a, b), the focus of the study is on these two oxidizing agents.

The aims of this work, therefore, are: (i) to elucidate the pertinent processes in South-Moravian lignite treatment; (ii) to test the obtained products from both chemical and physico-chemical perspectives; and (iii) to assess the influence of regeneration on their biological activity *in vitro*.

## Materials and methods

### Lignite regeneration; extraction and preparation of humates

All the chemicals were obtained from LachNer Ltd., Neratovice, Penta Ltd., Chrudim, (Czech Republic) and SigmaAldrich Co., Steinheim (Germany). South Moravian lignite (kindly provided by Lignit Hodonín Ltd., Mikulčice, Czech Republic) was regenerated with two oxidizing agents – nitric acid (abbreviated as N) and hydrogen peroxide (abbreviated as P) in concentrations of 10, 20, 30, 40, 50, 65 vol% and 5, 10, 20, 30 vol%, respectively. The lignite:oxidizing agent ratio was 1:10 w/w (50 g of parental lignite in 500 mL of regeneration agent). Lignite was treated for 30 minutes at a temperature around 30°C in a glass beaker. For the 50 and 65 vol% nitric acid treatment a round bottom flask with a reflux Allihn condenser and a cooling bath were used, since the regeneration reactions were strongly exothermic. The mixture was filtered and the filtration cake of treated lignite was used for extraction of regenerated humate. A quantity of 40 g of lignite was subjected to extraction *via* a slightly modified process published by Swift (1996). It involved an alkaline extraction with a mixture of 0.5 mol L^–1^ sodium hydroxide and 0.1 mol L^–1^ sodium pyrophosphate. The lignite:agent ratio was 1:10 w/w. Separation was achieved by centrifugation for 15 min at 15°C and 4000 rpm with Rotina 46 R centrifuge (Andreas Hettich Ltd., Tuttingen, Germany), and precipitation by addition of concentrated hydrochloric acid. Further purification included removal of silicate residues with 5 vol% hydrofluoric acid and dialysis against deionized water through a SpectraPor 1000 Da cutoff dialysis membrane made of regenerated cellulose (Spectrum Labs Inc., Rancho Dominguez, CA, U.S.A.).

After extraction and dialysis, each humic sample was divided into two parts. The first part was freeze dried as such, yielding solid humic acid of low solubility. The second part was titrated with 0.5 mol∙L^–1^ potassium hydroxide to a pH of 7.2, using a TitroLine Alpha Plus automated titrator (Schott Inc., Mainz, Germany), and then freeze dried. This yielded water soluble potassium humate. Freeze drying was carried out with Freezone 4.5 freeze dryer at -50°C and 120–140 mPa (Labconco Corp., Kansas City, MO, U.S.A.). The products obtained were crushed in an agate mortar, weighed, and stored in sealed vials in a dry, dark location. The samples descriptions are summarized in Table 1.

**Table 1:** **Sample descriptions**

Sample abbreviation	Sample description
HA	Humic acid from parental lignite.
RHA10N–RHA65N	Humic acid from lignite regenerated with nitric acid solution (N), the number means vol% concentration.
RHA5P–RHA30P	Humic acid from lignite regenerated with hydrogen peroxide (P) solution, the number means vol% concentration.
KHA	Potassium humate salt from parental lignite.
KRHA10N–KRHA65N	Potassium humate salt from lignite regenerated with nitric acid solution (N), the number means vol% concentration.
KRHA10N–KRHA65N	Potassium humate salt from lignite regenerated with hydrogen peroxide (P) solution, the number means vol% concentration.

### Solid state analyses of humic acids

#### Elemental analysis

Elemental analyses of HA and RHA samples were conducted employing a Perkin Elmer 2400 CHNS/O Elemental Analyzer. The oxygen percentage was calculated as a difference between the sample weight and the C, H, and N content, taking into account the moisture and ash contents determined by thermogravimetry (see below).

#### Thermogravimetry

The influence of regeneration on the thermooxidative stability of humics products was assessed by thermogravimetry. Prior to the analysis, the samples were dried for two weeks over sodium hydroxide, and then analyzed with a Q5000 IR TGA instrument (TA Instruments Inc., New Castle, DE, U.S.A.). The 100-μL platinum pans of the apparatus were used as sample holders, and the analysis was carried out using a 10°C∙min^–1^ temperature ramp from room temperature (RT) to 650°C, under a 50 mL∙min^–1^ flux of dry air.

#### Fourier transform infrared spectrometry

Infrared spectra were obtained using potassium bromide pellets technique, in which 1 mg of oven dried (105°C, 3 h) humic material (HA and RHA) was mixed with 200 mg of dried FTIR grade KBr. Spectra were measured with a Thermo Nicolet iS10 infrared spectrometer (Thermo Fisher Scientific Inc., Waltham, MA, U.S.A.). The instrument was set up with a resolution of 8 cm^–1^ and 64 scans per analysis. The spectra were processed using the Nicolet Omnic 8 software.

### Liquid state analyses of humates

#### Dynamic light scattering

Dynamic light scattering measurements were obtained with a N4 Plus Submicron Particle Sizer equipped with He-Ne red laser of wavelength of 632.8 nm (Coulter Corp., Miami, FL, U.S.A.), calibrated using latex beads from the producer. Sample solutions of 250 mg∙L^–1^ were prepared in water and filtered through GN 0.2 μm filters (Millipore Corp., Billerica, MA). Unimodal Analysis Mode was used with a detection angle of 90°. Measurements were taken in 10 runs of 300 s each, at 25°C in a 1-cm quartz cuvette. Outlying values were excluded according to the Dean-Dixon test (Dean and Dixon 1951).

#### High performance size exclusion chromatography

High performance size exclusion chromatography (HPSEC) experiments were performed using an Ultimate 3000 Standard Chromatography Station (Dionex Inc., Sunnyvale, CA, U.S.A.), equipped with a BioSep S2000 600 × 7.8 mm column, and a BioSep Guard pre-column with a 0.2-μm stainless steel inlet filter (Phenomenex Inc., Torrance, CA, U.S.A.). The column was thermostated at 25°C, and a diode array detector was employed. The eluent was a 50 mmol∙L^–1^ solution of NaH_2_PO_4_∙H_2_O in MilliQ water adjusted to pH 7 by means of 1 M NaOH solution. The flow rate was set at 0.6 mL∙min^–1^. Samples were prepared as 0.6 mg∙mL^–1^ solutions of potassium humate dissolved in mobile phase, and 100 μL of the sample was injected. The calibration of the column was performed using poly(styrenesulphonate) standards of 194.2, 145, 32.9, 14.9, 6.53 and 0.91 kDa mass (Polymer Standards Service Ltd., Mainz, Germany). Calibration curves and results were obtained using Dionex Chromeleon, Microsoft Excel, and OriginLab software. The weight averaged (*M*_W_) and number averaged (*M*_N_) molecular weights of the humates were calculated according to the Mori and Barth (1999). For the purpose of comparison, an 1100 Series Chromatography Station (Agilent Inc., Santa Clara, CA, U.S.A.) equipped with quaternary pump and an RI detector was used with the same column and under identical conditions. Calibration was carried out using polysaccharide standards (of 404, 212, 112, 47.3, 5.9, 0.667 kDa mass, same purveyor).

#### Humate hydration

In order to determine the amount of water of hydration bound by the humics, a high resolution ultrasonic spectrometer (HRUS 102, Sonas Technologies Ltd., Dublin, Ireland) was employed. Measurements of ultrasonic velocity for each sample were conducted in two independent quartz cells, stirred at 600 rpm with bottom and top stirrers. The system was kept at 25.00 ± 0.02°C, using a water bath. The instrument was calibrated with deionized water at 25.00 ± 0.02°C. Ultrasonic experiments are based on the determination of difference in ultrasonic velocities in cell 1 (1 mL of sample solution) and cell 2 (1 mL of pure water) (the U12 value). The concentration of the sample (potassium humate) was always 250 mg∙L^–1^, measurements of ultrasonic velocity were repeated three times at three different frequencies (5478, 7850 and 12196 kHz).

High resolution density measurements were performed for the same solutions, in triplicates, with a DMA 4500 density meter (Anton Paar Ltd., Graz, Austria).

Resulting compressibility (*β*) and hydration of the humates were calculated according to equations published by Davies et al. (1982), which assumes that the compressibility of hydration water is much lower than that of non-interacting solvent and of the humic substances themselves.

### Biological activity

The common maize *Zea mays* CEKLAD 235 species (Oseva Bzenec Ltd., Czech Republic) was selected in the biological studies as treated seed for its universality, easy availability, high durability, and good germination percentage. The seeds were treated for 5 min in a 50 mM sodium hypochlorite (NaClO) solution and then washed and immersed in deionized water for 4 h to precondition them for germination. The germination was conducted in wet Tork Wiper 430 paper laboratory towels (Thermo Fisher Scientific Ltd., Pardubice, Czech Republic), into which the seeds were rolled, separated by approximately 3 cm gaps. The paper rolls with seeds were put in a glass beaker containing deionized water and left to germinate for 2 days in the dark at 28 ± 2°C, employing a BT-120 Biological Thermostat (Laboratorní přístroje Praha Ltd., Prague, Czech Republic).

**Figure 1:**
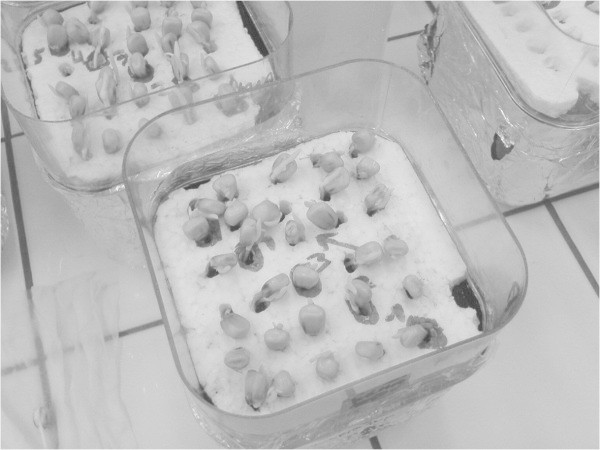
**Placing the germs in testing polystyrene containers, with 30 germs per container and each 5 of them placed in a marked position on a floating styrofoam bed to be observed later for root length and division.**

Selected germs (2–4 cm) were planted in polystyrene containers, with 30 germs per container and each placed in a marked position on a floating styrofoam bed to allow the observation of root length and division (see Figure 1). A solution of 2 mM CaCl_2_, without nutrients added, was employed as a control (Zandonadi et al. 2007). The humate sample solutions (1 L per vessel) contained 2 mM CaCl_2_ and 40 mg∙L^–1^ potassium humate (Antošová et al. 2008). For the purpose of comparison, a commercial nitrophenolate growth promoter (AtonikPro, ArystaLifeScience, Czech Republic) was also included in the testing regimen. This solution contained 0.4 vol% of the product (manufacturer’s recommendation) and 2 mmol∙L^–1^ solution of CaCl_2_. A combination solution containing 1 L of 2 mM CaCl_2_, 0.4 vol% of AtonikPro, and 40 mg∙L^–1^ of KRHA10N (potassium humate from lignite regenerated with 10 vol% HNO_3_) humate was prepared too, as well as a similar solution with KRHA30P (potassium humate from lignite regenerated with 30 vol% H_2_O_2_). The growth experiments were conducted in a BT 120 device for 5 days at 25 ± 2°C. A cycle of twelve hours of simulated daylight per day was produced with a NanoLight 9 W lamp of 600 lumen (Dennerle Ltd., Vinningen, Germany), placed 20–30 cm from the plants. This resulted in a light intensity of 1500–2500 lux. Remaining 12 h of the experiment proceeded in the dark. All the containers were continually aerated by means of a Precision Aquarium Pump of 4 W power and 275 L∙h^–1^ flow rate (Sera Ltd., Hainsberg, Germany). All the experiments were performed in duplicate. Germination tests were not performed, since they have been already studied for the humic acid extracted from South-Moravian lignite by Šerá and Novák (2011), when germination stimulating effects have been proven for this humic acid even on non-cultural plant of Lamb’s Quarters (*Chenopodium album*). Similarly, the stress tests on the plants have not been applied, since the plants seemed to present uniform leave numbers, lengths and areas and these would be far beyond the scope of this work.

### Biological activity assessment

The germinated seeds and grown plants were weighed and the differences were recorded in terms of total mass increment. In addition, the lengths of the roots of five marked germs/plants were measured before and after the growing experiment, and the differences in root growth were recorded. The roots of these five plants were also scanned with a Perfection 2480 Photo Scanner (Epson Deutschland Ltd., Meersbuch, Germany) against a black background. The resulting images (300 × 300 dpi) were captured by means of HarFA (Harmonic and Fractal image Analyzer) software (http://www.fch.vutbr.cz/lectures/imagesci) (Zmeškal et al. 2001), which was previously shown to be suitable for the evaluation of images from biological experiments (Tománková et al. 2006) and neuroscience (Wu et al. 2010). The scanned images were saved as bitmaps and subjected to 2D Wavelet Analysis. Overall results of biological activity were assessed by means of R Environment for Statistical Computing software (http://www.R-project.org) (R Development Core Team 2012). The root growth increments for the particular 15 samples were statistically compared using a one-way analysis of variance (ANOVA). Post-hoc multiple comparisons were conducted using Tukey’s HSD (Honestly Significant Difference) test. The assumptions were evaluated using Levene’s test and the Shapiro-Wilk’s test. Slight discrepancies vis-à-vis the normality assumptions were disregarded, as they were considered to have minimal impact on the results.

Rather than plants stress tests, we focused on the determination of sugars and protein in the seedlings. After the image analysis, the seedlings were dried in a laboratory dryer for three days at 60°C. The total dry mass was divided for determination of sugars and proteins. The sugar content was determined polarimetrically using the customary Ewers polarimetric method (Kennedy et al. 1995). The proteins were determined by the Kjeldahl method (McClements 2001) for the determination of total nitrogen. An automatic Kjeldahl analyzer Kjel-Tec™ 2100 (FOSS Inc., Hillerød, Denmark) was used.

## Results and discussion

### Lignite treatment and humate regeneration

Table 2 shows the yields of humate regenerated from lignite. Regeneration resulted in higher yields, when performed with nitric acid, with the exception of 10 vol% HNO_3_. Regarding the hydrogen peroxide treated lignites, only the 20P sample gave a greater HA yield than direct extraction from parental lignite.

**Table 2:** **Results of solid humic acids analyses**

Sample		Yield	Elemental analysis							Thermogravimetry			FTIR Relative peak intensity ratios		
			C	H	N	O	H/C	N/C	O/C	Humidity	1st onset	Ash	Aliph./Arom.	Arom./O– groups	Arom./Amid.
		[wt%]	[at%]	[at%]	[at%]	[at%]	[1]	[1]	[1]	[wt%]	[°C]	[wt%]	[1]	[1]	[1]
HA		10.7	43.5	37.0	0.9	18.6	0.85	0.02	0.43	5.8	202.8	0.9	1.24	0.93	1.08
RHA samples	10 N	11.0	40.0	45.8	0.6	13.6	1.15	0.02	0.34	5.4	226.6	0.5	1.23	0.99	1.08
	20 N	28.4	38.2	42.7	2.1	16.9	1.12	0.06	0.44	5.8	134.8	0.3	1.20	0.96	0.98
	30 N	24.6	37.9	42.0	2.4	17.7	1.11	0.06	0.47	5.8	162.4	0.4	1.24	0.96	0.95
	40 N	18.8	38.3	40.9	2.9	17.8	1.07	0.08	0.46	4.3	153.1	0.4	1.13	0.97	0.94
	50 N	34.7	38.4	41.1	2.7	17.8	1.07	0.07	0.46	4.9	152.6	0.5	1.20	0.97	0.93
	65 N	35.5	36.7	43.5	2.9	17.0	1.19	0.08	0.46	5.2	169.7	0.5	1.18	0.98	0.90
	5P	10.1	41.6	43.4	0.9	14.1	1.04	0.02	0.34	6.2	200.1	0.3	1.22	0.98	1.18
	10P	9.1	40.2	44.5	0.8	14.5	1.11	0.02	0.36	6.2	192.2	0.3	1.25	0.98	1.12
	20P	13.6	39.2	45.1	0.7	15.0	1.15	0.02	0.38	4.7	169.8	0.3	1.31	0.98	1.14
	30P	7.0	38.9	46.6	0.4	14.1	1.20	0.01	0.36	3.7	204.0	0.3	1.24	0.99	1.07

### Solid state analyses of humic acids

Results of the elemental analysis are presented in Table 2. A notable feature of the lignite treatment was the variation in nitrogen content of the regenerated humic acids – especially through treatment with 20–40 vol% HNO_3_. The increase in nitrogen content in samples RHA20N to RHA40N, and the slight decrease in RHA50N and RHA65N confirmed the previous observation about the concentration effect on the yield (Kučerík et al. 2003). The HNO_3_ treatment resulted in producing of nitrogen enriched humic substances, with the exception RHA10N. This is in contrast to the results reported previously by Čtvrtníčková et al. (2011) and Vlčková et al. (2009) who reported an N increase in the regenerated products obtained in a similar process. The reason for this discrepancy is not entirely clear; it might be related to variation of humics from batch to batch within the same source. The HNO_3_ treated samples showed also a higher oxygen content and larger O/C ratio, indicating an increase in the number of oxygen bearing groups. Comparison with humic standards provided by International Humic Substances Society (IHSS 2013) shows that the samples from the nitric acid treated lignite were, in terms of N content, more reminiscent of Elliot Soil HA than of Leonardite HA. HA obtained from H_2_O_2_ treated lignite had lower oxygen content and O/C ratio, higher H content and H/C ratio and lower N content. Since the O content was calculated as a difference, the determined value may include traces of S and P.

**Figure 2:**
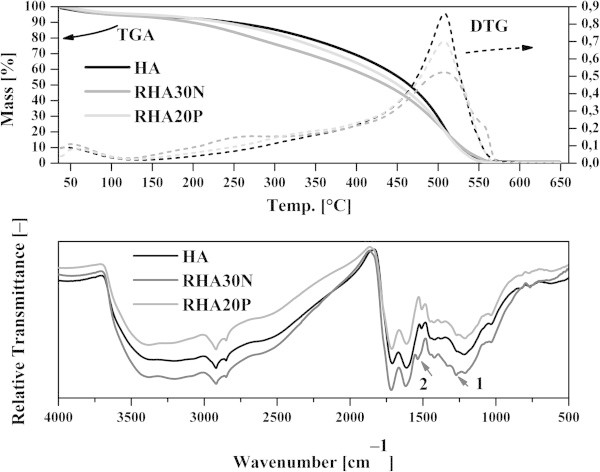
**Thermogravimetrical (TGA) curves (line – TGA, dashed – DTG [1st TGA derivative]) and Fourier transform infrared (FTIR) spectra of selected solid samples (HA – black, RHA30N – grey, RHA20P – light grey).**

The regenerated humic acids were characterized by thermogravimetry and results were compared with humics extracted from parental lignite (parental HA). Selected thermo-oxidative curves are shown in Figure 2. These decomposition curves were comparable to results obtained by other groups and they were typical for lignitic humic substances (Gonet and Cieslewicz 1998). The parameters extracted from TGA curves are summarized in Table 2. It is worth to mention that the onset temperatures represent a measure of the thermo-oxidative stability. The rate of material degradation is clear from the DTG curves in Figure 2. The first peak of the DTG curve (150–350°C), which is generally ascribed to the degradation of the labile part of humics, was steeper and associated with larger mass loss in the RHA samples, while the second, higher and narrower DTG peak (450–550°C, assigned to stable structures) was generally lower. The lower onset temperatures and steeper degradation curves of regenerated HA samples indicate a relative enhancement of aliphatic structures and polar functionalities, which are generally less thermo-oxidative stable than aromatic moieties (Plante et al. 2009). These findings are in agreement with negative correlations between the H/C and N/C ratios and the thermal stability reported by Gonet and Cieslewicz (1998).

FTIR spectra of all HA and RHA materials were comparable to those reported in the literature (Stevenson 1994), examples are reported in Figure 2. The incorporating of nitrogen atoms into the humic structure by the HNO_3_ lignite treatment was confirmed by 1540–1520 cm^–1^ peaks due to the Ar–NH/NO_2_ moiety (RHA20N–RHA65N samples). The strong effect of concentrated HNO_3_ was corroborated by the 1333 cm^–1^ peak of CO–NH and NO_2_ (in RHA40N–RHA65N samples). In the RHA20P and RHA30P samples, the 1269 cm^–1^ peak of aromatic/R–COOR moieties was also present. To distinguish between parental HA and the RHA samples, we calculated the intensity ratio of the 2930 and 1610 cm^–1^ absorption bands (i.e. aliphatic/aromatic ratio) (Table 2). To assess the lignite modification by HNO_3_, we determined also the intensity ratios of the absorption bands at 1550–1520 cm^–1^ (amidic) and 1610 cm^–1^ (aromatic). All the regenerated samples gave a slightly higher aliphatic/aromatic ratio than parental HA. The highest value was obtained for RHA40N, followed by the samples RHA65N, RHA50N, RHA20N and RHA5P. The lowest aliphatic content (but still higher than the HA sample) appeared in the RHA30P sample.

### Liquid state analyses

**Figure 3:**
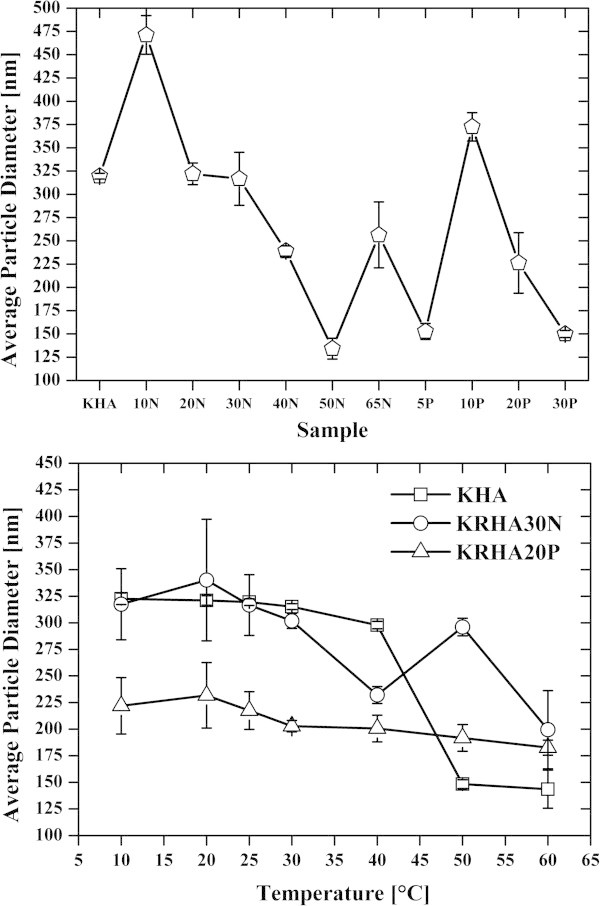
**Dynamic light scattering (DLS) results of humate solutions, temperature variability of aggregate size (up; pentagons), aggregate sizes for different samples at 25°C (down; squares – KHA, circles – KRHA30N, triangles – KRHA20P).**

Dynamic light scattering measurements (DLS) showed the presence of large dimension particles, most likely aggregates of diameters varied in the range 100–500 nm (Figure 3). In view of the pseudomicellar model of humic aggregation and when compared to the DLS results of Palmer and von Wandruszka (2001), the humate samples showed abnormal behavior. The KHA, KRHA20N, KRHA30N and KRHA65N samples had an aggregate size of 275–325 nm at 25°C (Figure 3) which is comparable to previously found values, for the Leonardite humic acid and Nordic aquatic IHSS humics (Palmer and von Wandruszka 2001). On the other hand, KRHA50N, KRHA5P and KRHA30P samples had smaller aggregate sizes of 125–175 nm, comparable to Summit Hill humic acid and Suwannee River humic acid. From this perspective, the regeneration brought more soil-humic-acid-like behavior to the samples. The fluctuation of the aggregate sizes with increasing temperature can be ascribed to the amphiphilic nature of humic acids and the reduction of their hydration sphere (Drastík et al. 2013).

**Figure 4:**
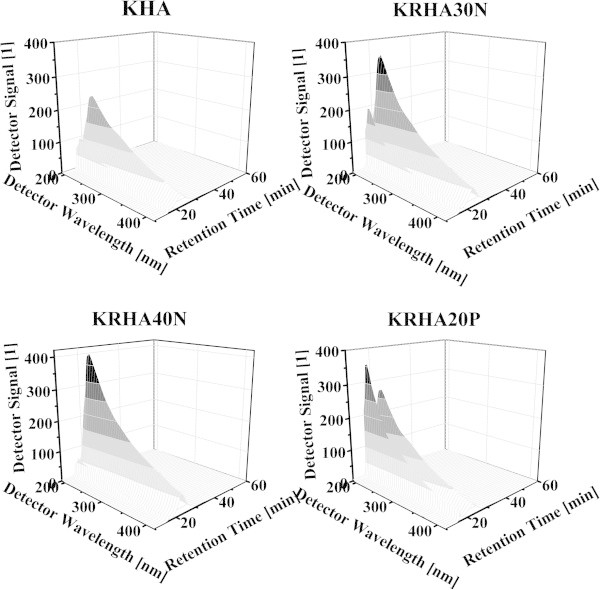
**3D high performance size exclusion chromatography (HPSEC) chromatograms of selected humate samples.**

**Figure 5:**
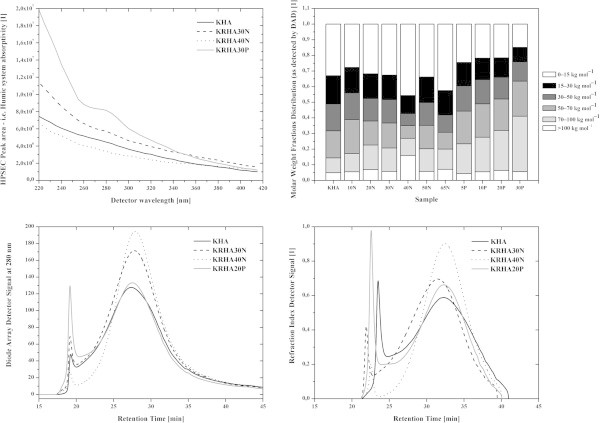
**High performance size exclusion chromatography with Diode array detection (HPSEC DAD) peak area of selected humate samples (upper left; line – KHA, dash – KRHA30N, dot – KRHA40N, grey – KRHA30P), molar weight distribution as detected by DAD at 280 nm in humate samples (upper right; greyshades according to the legend), DAD detector signal at 280 nm (lower left) and RID signal (lower right; for both the lower charts: line – KHA, dash – KRHA30N, dot – KRHA40N, grey – KRHA20P).**

High performance size exclusion chromatography (HPSEC) experiments were conducted with refractive index detector (RID) and diode array detector (DAD) in order to avoid the underestimation of a variety of molecular fractions absorbing in the visible range. All measurements produced a bimodal distribution (Figures 4 and 5). The sharp peak with a retention time (RT) of 18 min was attributed to the total exclusion of the largest solute components (aggregates or chain segments, composed mainly of hydrophobic alkyl chains with no fluorescence or UV absorption). Smaller species were eluted subsequently giving a peak between 20 and 45 min, ascribed to the shorter conjugated alkenes and strongly fluorescing or UV absorbing aromatics (Conte et al. 2007, von Wandruszka et al. 1999). HPSEC utilizing RID gave similar results for all samples, with only slight differences in RT and overall peak shape. The great differences were found between potassium humates obtained from parental and treated lignites, the HNO_3_ treatment of lignite caused an increased unsaturated content in the humates. The KRHA40N and KRHA65N samples showed fewer long chains, while the samples obtained from peroxidized lignite contained a significantly higher proportion of this fraction. The DAD showed a decrease in humate absorbance with the increasing wavelength (Figure 5), while the combined DAD peak area at a particular retention time and wavelength reflected the content of specific humic components. The peak areas were plotted against the respective wavelengths, emphasizing the differences between investigated samples (Figures 4 and 5). The regeneration, except for KRHA40N, increased the chromophoric content of the humic matter. This indicates that the oxidation of lignite had a major impact on the aliphatic moieties, while affecting the aromatic ones to a lesser extent. The irregular behavior of KRHA40N was probably due to inhomogeneities in the lignite fraction used in the preparation of this sample. The observed differences between *M*_W_ and *M*_N_ among the humate samples (Table 3) indicated a high degree of polydispersity and potentially chemical heterogeneity in the materials. The RID results indicate that nitric acid treatment decreased the molecular mass of extracted humic acids, while peroxide had the opposite effect. The *M*_W_/*M*_N_ ratio was used as an indicator of the system polydispersity (PDI), and results showed that higher concentrations of oxidizing agent caused a narrower mass distribution while lower concentration caused a slight increase (RID). This was in line with recent results indicating that the oxidation of lignite proceeds in several steps depending on the oxidation time and/or strength of oxidizing agent (Kučerík et al. 2008a, b). The integrated DAD spectral peak areas, after division into 6 intervals of mass distribution, clearly showed the differences between the samples (Figure 5).

**Table 3:** **Results of high performance size exclusion chromatography as detected by DAD and RID**

Sample		***M***_N_(280 nm DAD) [g∙mol^–1^]	***M***_W_(280 nm DAD) [g∙mol^–1^]	PDI (280 nm DAD) [1]	***M***_N_(RID) [g∙mol^–1^]	***M***_W_(RID) [g∙mol^–1^]	PDI (RID) [1]
	KHA	7 879	89 733	11.39	6 716	52 915	7.88
KRHA samples	10 N	8 954	107 105	11.96	6 474	52 180	8.06
	20 N	8 715	91 669	10.52	5 805	36 275	6.25
	30 N	8 355	77 605	9.29	5 816	34 420	5.92
	40 N	5 797	198 983	34.33	5 240	21 900	4.18
	50 N	7 959	73 161	9.19	6 153	36 435	5.92
	65 N	6 408	87 198	13.61	6 145	24 755	4.03
	5P	10 782	75 495	7.00	7 361	49 975	6.79
	10P	11 914	86 120	7.23	6 817	55 300	8.11
	20P	11 934	86 066	7.21	6 801	56 390	8.29
	30P	14 643	83 739	5.72	7 766	82 440	10.62

**Figure 6:**
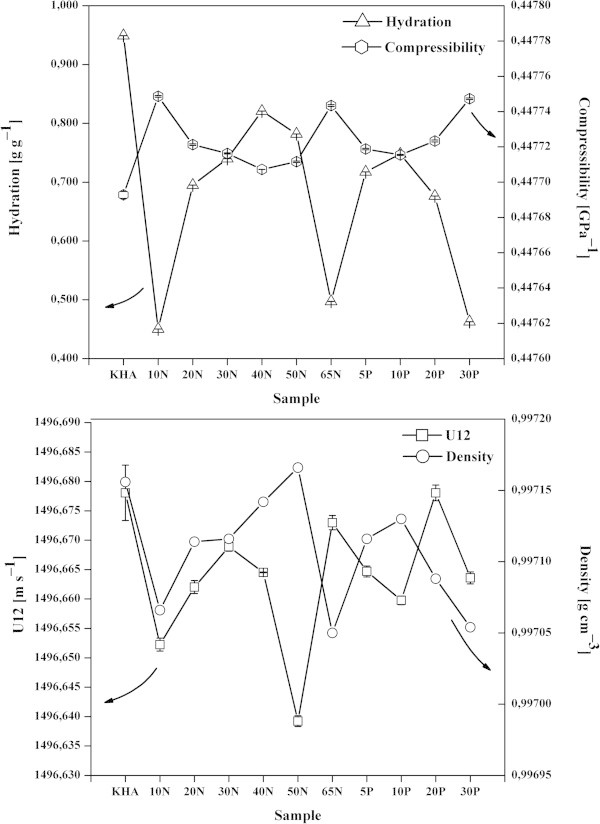
**Up: hydration (left axis, triangles) and compressibility (right axis, hexagons) chart; down: ultrasound velocity (U12) (left axis, squares) and density (right axis, circles) chart.**

The hydration of humates was studied by means of high resolution ultrasonic velocimetry and density measurements. Figure 6 presents graphs showing the variation of hydration, compressibility, ultrasound velocity, and density in the different materials used in this study. The hydration values of the humates (*e.g.* KHA, KRHA30N, KRHA40N, KRHA50N, KRHA5P, and KRHA10P) were found to lie relatively high, i.e. between 0.45 and 0.95 grams of water per gram of humate (Figure 6), similar in magnitude to those found for hyaluronan, which is considered to be the most hydrated polysaccharide (Davies et al. 1982; Průšová et al. 2010). This was probably a consequence the high porosity of the humic aggregates (Drastík et al. 2013; Kučerík et al. 2012): the water trapped in their interior contributed to the measured hydration value.

### Biological activity

**Figure 7:**
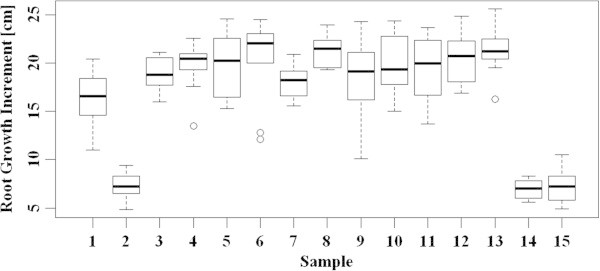
**Biological activity - root growth increment boxplot.** 1 – Control, 2 – AtonikPro, 3 – KHA, 4 – KHA10N, 5 – KHA20N, 6 – KHA30N, 7 – KHA40N, 8 – KHA50N, 9 – KHA65N, 10 – KHA5P, 11 – KHA10P, 12 – KHA20P, 13 – KHA30P, 14 – KHA10N + AtonikPro, 15 – KHA30P + AtonikPro.

**Figure 8:**
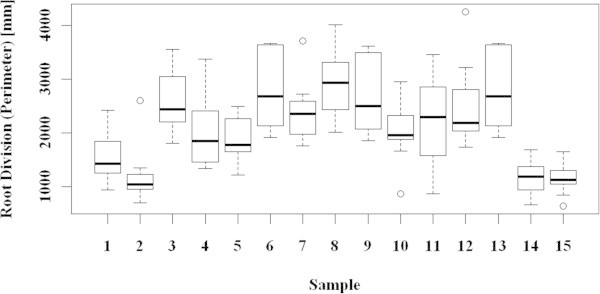
**Biological activity - root division (K[BW] – the root fractal measure) boxplot.** 1 – Control, 2 – AtonikPro, 3 – KHA, 4 – KHA10N, 5 – KHA20N, 6 – KHA30N, 7 – KHA40N, 8 – KHA50N, 9 – KHA65N, 10 – KHA5P, 11 – KHA10P, 12 – KHA20P, 13 – KHA30P, 14 – KHA10N + AtonikPro, 15 – KHA30P + AtonikPro.

It was found that the differences between the samples were statistically highly significant (ANOVA *p*-value < 0.001). The results are shown in Figure 7 (boxplot for root growth increment) and Figure 8 (boxplot for root division), and are further summarized in Table 4. Treatments with AtonikPro, AtonikPro + KRHA10N, and AtonikPro + KRHA30P led to significantly lower root growth increments than those of the control group (all with *p*-values <0.001). These treatments also presented decreasing effects on root growth compared to the remaining 11 samples (*p*-values <0.001). Plants grown in an AtonikPro solution showed substantially shorter and less divided roots, but their main roots and lateral roots were significantly thicker and presented the highest mass increments. This may have been caused by the nitrophenolate nature of AtonikPro, which is designed to increase the yield of crop plants (Hejnák 2010). In contrast, KRHA50N, KRHA20P, and KRHA30P yielded significantly larger root growth increments compared to the Control (*p*-values 0.011, 0.043 and 0.011 respectively). Differences among these three samples were comparable (statistically not significant). The differences between the remaining samples (KRHA10N, KRHA20N, KRHA30N, KRHA40N, KRHA65N, KRHA5P, KRHA10P) and the control group were found not to be statistically significant, despite the fact that the means of sample root growth increments were larger compared to the Control. This may be due to the relatively small number of observations per sample (10), which reduced the reliability of the tests.

**Table 4:** **Biological activity results of humic samples and AtonikPro (Root growth increment, plant mass increment and root division (Perimeter); averages with respective standard deviations**

	Sample	Number	Concentration	Root growth increment (2 × 5 roots)	Mass increment (2 × 30 plants)	K[BW] – root division (Perimeter) (2 × 5 roots)	Starch content (30 plants dry mass)	Protein content (30 plants dry mass)
				[cm]	[g]	[mm]	[wt%]	[wt%]
	Control	1	–	16 ± 3	27 ± 0.01	1524 ± 5	32	11
	AtonikPro	2	0.04 vol%	7 ± 1	29 ± 6	1180 ± 6	35	12
	KHA	3	40 mg L^–1^	19 ± 2	30 ± 6	2573 ± 6	34	11
KRHA samples	10 N	4	40 mg L^–1^	20 ± 3	29 ± 0.4	2158 ± 8	34	12
	20 N	5	40 mg L^–1^	20 ± 3	28 ± 5	1881 ± 4	32	12
	30 N	6	40 mg L^–1^	20 ± 4	25 ± 2	2792 ± 6	32	12
	40 N	7	40 mg L^–1^	18 ± 2	26 ± 0.3	2405 ± 6	29	11
	50 N	8	40 mg L^–1^	21 ± 2	26 ± 2	2944 ± 6	32	11
	65 N	9	40 mg L^–1^	19 ± 4	29 ± 9	2663 ± 7	34	12
	5P	10	40 mg L^–1^	20 ± 3	31 ± 3	2064 ± 6	33	13
	10P	11	40 mg L^–1^	19 ± 4	27 ± 0.5	2253 ± 9	33	13
	20P	12	40 mg L^–1^	21 ± 3	30 ± 3	2477 ± 8	32	12
	30P	13	40 mg L^–1^	21 ± 2	31 ± 5	2792 ± 7	31	11
	KRHA10N + AtonikPro	14	40 mg L^–1^+ 0.04 vol%	7 ± 1	38 ± 7	1181 ± 3	32	12
	KRHA30P + AtonikPro	15	40 mg L^–1^+ 0.04 vol%	7 ± 2	32 ± 2	1144 ± 3	32	11

The root divisions for the 15 samples were statistically compared in the same way and the differences among the samples were again found to be highly significant. The treatments KHA, KRHA30N, KRHA50N, KRHA65N, and KRHA30P lead to significantly larger root divisions (*p*-values 0.017, <0.001,<0.001, <0.006 and <0.001 respectively) compared to the control group. Furthermore, the results for KRHA30P were only borderline non-significant (*p*-value 0.051), and could also be included in the previous group. Finally, the same considerations regarding the test results applied here. The results for KHA and other treatments (except for AtonikPro and Control) did not differ significantly, even though the sample means and medians varied. The highest mass increment was observed in the plants grown in the mixture of 40 mg∙L^–1^ KRHA10N and 0.04 vol% of AtonikPro (sample 13).

The treatment of lignite had an effect on the starch and proteins content of the maize roots. Slightly higher starch contents were found in plants grown in AtonikPro, KHA, KRHA10N and KRHA65N solutions, while the use of AtonikPro, KRHA5P and KRHA10P led to increased protein content (see Table 3 for details).

All of the tested humates exhibited some degree of biological activity with regard to stimulating the growth of maize plants. Materials in both lower and higher molecular weight ranges (e.g. KRHA50N and KRHA30P, respectively) gave statistically significant positive results. Works by Canellas et al. (2002) or Zandonadi et al. (2007) have shown that chemical composition, rather than molecular weight distribution, is the prime factor in growth stimulation by humates. Most of the regenerated humates generally produced higher root growth (Figure 7) and higher root division (Figure 8), than native humate (KHA) extracted from the parental lignite, it should be noted, however, that neither root growth, nor root division showed enhancements that were statistically significant in all ten regenerated materials, as compared to native KHA. This is in line with the prediction by Vlčková et al. (2009) that oxidation of parental lignite leads to higher biological activity of the resulting humate product. Although the details of the oxidative mechanism are not known yet, it may be suggested that the introduction of additional carboxyl, hydroxyl, amino, and nitro groups play a role. As for root division, higher median values (relative to KHA) were observed for KRHA30N, KRHA50N and KRHA30P.

Table 4 shows that humates regenerated from lignite with nitric acid had positive effects on the starch content of the plants, while selected hydrogen peroxide regenerated humates showed increases in protein content. AtonikPro gave positive results in both cases, presumably because of its nitrophenolate nature (Hejnák 2010).

### Principal component analysis and correlations

**Figure 9:**
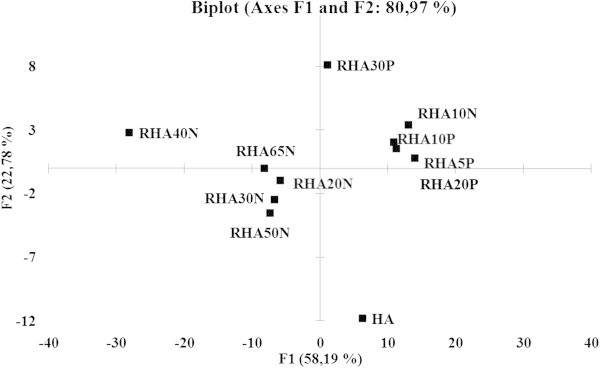
**Principal component analysis biplot of solid state experiments with humic acids (squares).**

Principal component analysis (PCA) is useful in the identification of patterns that highlight similarities and differences in data sets, especially in cases where the number of results in various measurement categories exceeds the number of samples (Meloun and Militký 2002; Smith 2002). In this work, PCA was applied to RHA and KRHA data generated by FTIR, elemental analysis, and TGA. The results were gathered in a scatter plot (Figure 9), which accounted for 81% of the variability and recognized 5 groups of samples. The first group comprised RHA10N, RHA10P, RHA5P and RHA20P, having a PC1 loading profile (data not shown) with positive values for the 1st onset TGA peak and IR absorption at 2930, 1520–1550, 1459, and 1422 cm^–1^. Their thermooxidative degradation started at a relatively high temperatures, and they had a high amide, C–O, –OH, aromatic C = C, and aliphatic content. Conversely, RHA65N, RHA20N, RHA30N and RHA50N were positioned along negative part of PC1 and were separated due to their low content of amide, aromatic C = C, C–O, O-H and aliphatic functional groups. At the same time, their values of N, (N + O)/C, and O/C were high. An extremely high amount of N and low amounts of aliphatic, aromatic C = C and amide functionalities caused RHA40N to be separated with a very negative PC score. The positive PC2 score involving only RHA30P indicated a significant contribution of H and H/C and pointed at low aromaticity. HA, lying along the negative PC2 axis, varied mainly due to its high char point of 650°C.

PCA of KRHA samples (data not shown) did not show significant variability in the data, with the first two PC values accounting for only 58% of data variability. The only significant differences were noted for KRHA40N, KRHA50N, and KRHA30P. The KRHA40N sample had the highest *M*_*w*_ and PDI (280 nm) values, while KRHA30P and KRHA50N showed more significant growth increments and root division.

The correlation coefficients shown in Table 4 confirmed that in terms of aliphatic, aromatic and amidic groups’ content, the FTIR results correlated well with the results for C, H, O and N content obtained from elemental analysis. The information from thermogravimetry, giving a negative correlation between O/N content and 1st TGA onset temperature, also confirmed that humic acid regeneration reduced the thermal stability of the materials (Gonet and Cieslewicz 1998). Generally, the more H was present in the humic sample, the lower the amount of ash produced in the thermogravimetric analysis became. Interestingly, the average aggregate particle size (AAPS) of the humates measured by DLS appeared to have no correlation with the molecular weights assessed by DAD and RID in the HPSEC separations. Likewise, no significant correlations of density and ultrasonic velocity with other parameters were observed. As for the HPSEC results, the *M*_N_ values obtained by means of the DAD detector (at 280 nm) were in a good agreement with those obtained by RID (*r* = 0.844, *p* < 0.05). Further, there was no correlation between *M*_N_ and *M*_W_ values obtained by DAD, while the correlation of both obtained by RID was significant and positive (*r* = 0.893, p < 0.05). This can be ascribed to the fact that the different detectors were sensitive to different moieties. It should be noted that the results of KRHA40N (mainly the DAD HPSEC *M*_W_ result) deviated strongly. No explanation is forthcoming for this circumstance, and it should probably be set aside as an artifact.

Inspection of the biological activities revealed that there was no correlation between root division and AAPS or root growth and AAPS. This indicates that these biological parameters were not affected by the aggregate size of the humates dissolved in the growth solution. The molecular weights, in particular *M*_N_ obtained with the DAD, did show a moderate correlation with root growth (*r* = 0.617, *p* < 0.05), while *M*_N_ values obtained with the RID were similarly correlated with plant mass (*r* = 0.772, *p* < 0.05). Positive correlations were also found for *M*_W_ obtained with the RID (*r* = 0.627, *p* < 0.05). The DAD results showed no correlation with mass increment (*M*_N_) for the entire set of 30 plants, while slightly negative borderline nonsignificant trend (*r* = -0.598, *p* < 0.1, *p* = 0.052) was found for *M*_W_. Positive correlations for molecular weight with root growth increment and plants mass increment were in line with previous findings (Canellas et al. 2010; Nardi et al. 2002). It also appeared that humates containing light absorbing groups induced root growth in *Zea mays*, with a correlation of *r* = 0.694, *p* < 0.05.

As for the relations between biological properties, root division did not correlate with root growth (Tables 4 and 5). Likewise, no positive correlations between the O and N content, or the aliphatic and amidic FTIR peak intensity ratios, with root division and root growth was found. Unexpectedly, we found the negative relationship between nitrogen content of the dissolved humates and the biological activity in terms of plants mass increment (*r = -*0.600 to -0.700, *p*’s <0.05. The correlations between particular molecular weights fractions, as detected by DAD at 280 nm, (Figure 5) and biological characteristics were assessed as well. The high (>100 kDa) and low molecular weight fractions showed no correlation or negative trend with the biological activity (e.g. *r* = -0.654, *p* < 0.05 for the root growth increment with the 0–15 kDa fraction). With respect to biological activity, the middle weight fractions of the humates (50–100 kDa) generally produced slightly positive correlations. Similar results were obtained for the nutritional properties of the plants (sugar and protein content). The various correlation values are summarized in Table 6.

**Table 5:** **Pearson’s correlation coefficients of chemical composition with humates’ biological activity**

	Elemental analysis [at%]							FTIR relative peak intensity ratios		
	C	H	N	O	H/C	N/C	O/C	Aliph./Arom.	Arom./O– groups	Arom./Amid.
K[BW] Root division	-0.267	-0.186	0.270	0.397	0.025	0.276	0.442	0.050	-0.120	0.421
Growth increment	-0.114	0.381	-0.285	-0.282	0.310	-0.281	-0.212	0.527	0.240	-0.197
Mass increment	0.475	0.290	-0.696	-0.528	-0.024	-0.686	-0.645	0.350	0.291	-0.631
Sugar content	0.391	0.044	-0.399	-0.247	-0.150	-0.389	-0.359	0.364	0.064	-0.323
Protein content	0.181	0.214	-0.183	-0.398	0.040	-0.180	-0.398	0.168	0.248	-0.377

**Table 6:** **Pearson’s correlation coefficients of molecular weight distribution fractions (detected by DAD at 280 nm) with humates’ biological activity**

	> 100 kg∙mol^–1^	70–100 kg∙mol^–1^	50–70 kg∙mol^–1^	30–50 kg∙mol^–1^	15–30 kg∙mol^–1^	0–15 kg∙mol^–1^
K[BW] Root division	-0.036	0.137	-0.200	-0.221	-0.118	0.100
Growth increment	-0.514	0.614	0.538	0.311	-0.199	-0.654
Mass increment	-0.371	0.420	0.502	0.206	-0.195	-0.491
Sugar content	-0.752	-0.173	0.422	0.713	0.628	-0.203
Protein content	-0.279	-0.082	0.271	0.353	0.230	-0.145

These results are in agreement with the findings by Vlčková et al. (2009) and the results of Canellas et al. (2010). Our findings do not agree with Nardi’s presumption (Nardi et al. 2002), that the greatest biological activity of humates lies with molecular weight fractions up to 3.5 kDa. Our work, however, supports later reports that showed the importance of higher molar weight humates (Canellas et al. 2010; Vlčková et al. 2009; Zandonadi et al. 2007).

## Conclusions

Lignite, in particular the South-Moravian lignite, represents a unique source of humic substances, but its exploitation as a raw material for environmental and agricultural scenarios is still limited. As demonstrated in this and earlier work, regeneration presents a way to increase the recovery of humic substances from lignite, and also allows for the tuning of their properties. The cost-benefit analysis was beyond the scope of this paper; however, the regeneration agents may be preferably recycled into the commercial fertilizer production. This may open a pathway to superior results with the use of these materials in agricultural applications. However, full control of these properties and biological activity still remains a challenge.
